# A topic modeling-based analysis of emerging mobility services for carbon emission reduction

**DOI:** 10.3389/fpubh.2025.1531401

**Published:** 2025-02-14

**Authors:** Yang Li, Yutian Lei, Zezhou Wu, Jiahao Wang, Tianjia Pei, Maxwell Fordjour Antwi-Afari

**Affiliations:** ^1^Guangdong Power Grid Corporation, Guangzhou, China; ^2^Energy Development Research Institute, CSG, Guangzhou, China; ^3^State Key Laboratory of Intelligent Geotechnics and Tunnelling, Shenzhen, China; ^4^Key Laboratory for Resilient Infrastructures of Coastal Cities, Ministry of Education, Shenzhen University, Shenzhen, China; ^5^Sino-Australia Joint Research Center in BIM and Smart Construction, Shenzhen University, Shenzhen, China; ^6^Department of Civil Engineering, College of Engineering and Physical Sciences, Aston University, Birmingham, United Kingdom

**Keywords:** emerging mobility services, carbon emissions, Latent Dirichlet Allocation, topic intensity, topic evolution

## Abstract

**Introduction:**

With rising urbanization and global climate change, sustainable city development has become an urgent challenge. Emerging mobility services provide innovative solutions for sustainable city governance, bridging the gap between transportation demand and supply, alleviating city travel and reducing carbon emissions. However, further investigation is needed to ascertain the specific roles and potential enhancements that emerging mobility services could contribute to reducing carbon emissions.

**Methods:**

In this study, a systematic search of the Web of Science Core Collection using relevant keywords yielded 431 articles. After screening, 225 articles were deemed relevant, meeting the following criteria: (1) the articles focused on emerging mobility services, and (2) they explored the relationship between these services, carbon emissions, and environmental impacts. These articles were then synthesized using the Potential Dirichlet Allocation Model.

**Results:**

This study identifies four key research questions using the Potential Dirichlet Allocation Model: “Emerging Mobility’s Environmental Impacts,” “Policy-Led Sustainable Mobility Services,” “User-Centric Mobility Services,” and “Cost-Benefit Analysis of Electrification.” Assessments of the theme strengths track their evolution over time, highlighting the increasing importance of policy-led development and user-centered optimization.

**Discussion:**

The study has shown that emerging mobility services have the potential to reduce carbon emissions; however, the extent of this impact varies by region, service type, and other factors. Policy strategies play a crucial role in promoting the development of new and emerging transport services. These findings contribute to the sustainable development of these services, the reduction of carbon emissions, and the improvement of urban living conditions.

## 1 Introduction

Rapidly increasing greenhouse gas emissions pose significant challenges to the global ecosystem and human health (1, 2). In the urban transportation sector, an excessive number of vehicles contribute to traffic congestion and environmental pollution (3). The carbon emissions of the transportation sector are significant, accounting for over 25% of global total carbon emissions (4). Therefore, achieving carbon emission reduction in the urban transportation industry is crucial for mitigating climate change (5). To address this pressing challenge, alleviate traffic congestion, and balance the demand for transportation services, many emerging mobility service models have emerged in the rapid development of mobile internet technology. These emerging mobility services include ride-hailing (6), ride-sourcing (7), ride-pooling (8), ride-sharing (9), carsharing (10), bike-sharing (11), e-scooters (12), mobility-as-a-service (13), and shared autonomous vehicles (14). While these emerging mobility services have significant potential to reduce carbon emissions, it is crucial to gain a deeper understanding of the actual impact of such services in the realm of carbon emissions and how to optimize them to their fullest potential.

In recent years, researchers have explored the relationship between emerging mobility services and sustainable transportation from various perspectives. Some researchers have used bibliometric methods to study trends in the development of emerging mobility services and sustainable transportation. Zhao et al. (15) utilized CiteSpace software to conduct a comprehensive review of literature published between 2000 and 2019 in the field of sustainable transportation, identifying research hotspots and proposing future directions. Trends in shared bicycle research were explored by Zhou et al. (16). These studies have adopted a bibliometric approach, which focuses more on the statistical analysis of the literature than on exploring the implicit thematic structure of the texts, and lacks sufficient depth in examining the research content of each study (17). Therefore, this study employs Latent Dirichlet Allocation (LDA), which allows for a deeper exploration of emerging mobility services.

This study employs a text mining approach. Unlike CiteSpace software, topic models can extract latent abstract topics from extensive text, thus organizing research trends. LDA is an unsupervised probabilistic generative model. This method can discover latent topics in a corpus, presenting the probability of topic occurrence in documents. LDA effectively captures the thematic characteristics of text and is widely applied in text topic identification research because it is not strictly limited by text length. Currently, LDA has been widely used in various fields. The introduction of LDA topic modeling significantly reduces the subjectivity in the literature classification process, allowing researchers to objectively discover latent topics and improve the way scholars think and interpret topics when dealing with textual data. The method offers significant advantages in transportation research. It effectively identifies potential research directions and issues, as well as predicts future shifts in transportation patterns through trend analysis (18). Moreover, the themes uncovered by LDA provide crucial guidance for planners and decision-makers in the transportation sector (19). In the context of emerging mobility services, LDA facilitates understanding of both the current state and future trends of these services, offering valuable insights for market stakeholders. Therefore, in this paper, LDA is adopted as the strategy for presenting topics.

This study employs LDA topic modeling to identify and analyze the research topics of emerging mobility services in the field of carbon emissions from 2015 to 2023. Based on this, the evolution of topics over time is calculated to gain a deeper understanding of their development trends and propose future research directions. The remainder of this paper is organized as follows: Section 2 introduces data collection and processing, the implementation of the LDA model, and the calculation of topic intensity. Section 3 presents the analysis results of the topics and their evolutionary trends. Finally, Section 4 provides the conclusion of the article.

## 2 Methods

This section outlines the research methodology, commencing with an overview of data collection (Section 2.1), followed by a description of data processing (Section 2.2). Subsequently, it expounds upon the application of topic modeling techniques (Section 2.3), culminating in an explanation of the computation of topic intensity values (Section 2.4).

**Figure 1:**
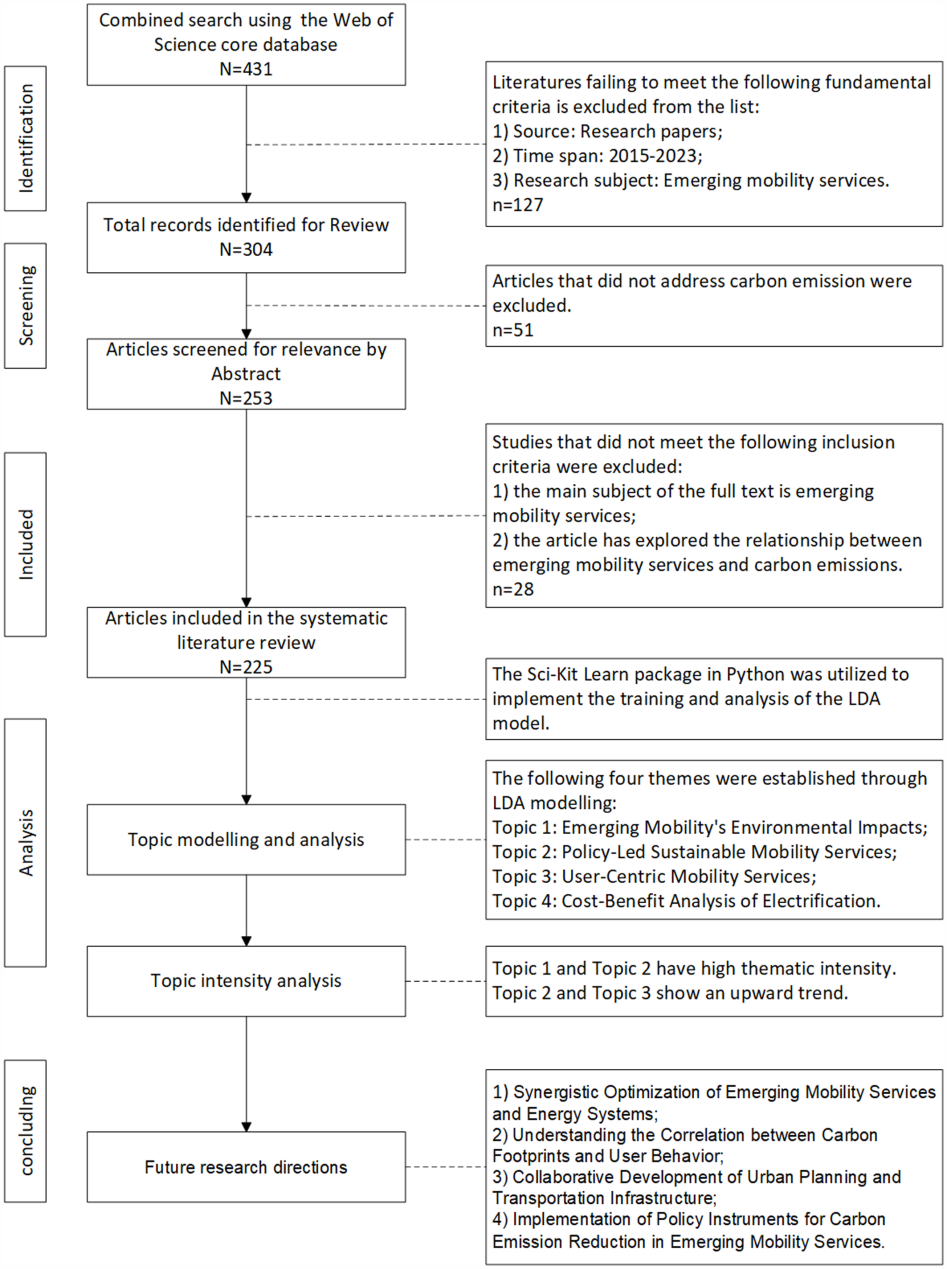
Research programme.

As depicted in Figure 1, this figure shows the implementation process and findings of this study.

### 2.1 Data collection

During the data collection phase of the study, the Web of Science core database served as the primary resource for literature retrieval. Publications indexed in the database are globally recognized, peer-reviewed, and considered to be of high quality. Given the focus on the study of emerging mobility services within the context of carbon emissions, systematic keyword searches were executed within the Web of Science core database. These keywords encompassed terms such as “ride-hailing,” “ride-sourcing,” “ride-pooling,” “ride-sharing,” “carsharing,” “bike-sharing,” “e-scooters,” “mobility-as-a-service,” “shared autonomous vehicles,” and “carbon emission.” A total of 431 articles were retrieved.

Subsequently, a screening process was carried out on the collected papers. Firstly, only articles with a time span of 2015–2023 were retained. Since the concept of emerging mobility services was introduced after 2015, the scope of this review begins from that year. 366 articles were retained in this step; then 62 collected papers and standards were excluded, leaving 304 research papers. These articles were then identified for relevance. By identifying titles, papers that were not relevant to emerging mobility services were eliminated, leaving 278 articles. Next, the abstracts of the articles were consulted and articles that did not address carbon emission or environmental impacts were excluded, leaving 253 articles. Finally, the 253 articles were read in depth and in detail, and those that met the following criteria were included in this study: (1) The focus of this article is the study of emerging mobility services; and (2) It explores the relationship between these services and carbon emissions, as well as their environmental impact. As a result of this selection process, 225 papers met these two criteria and were eligible for review.

**Figure 2:**
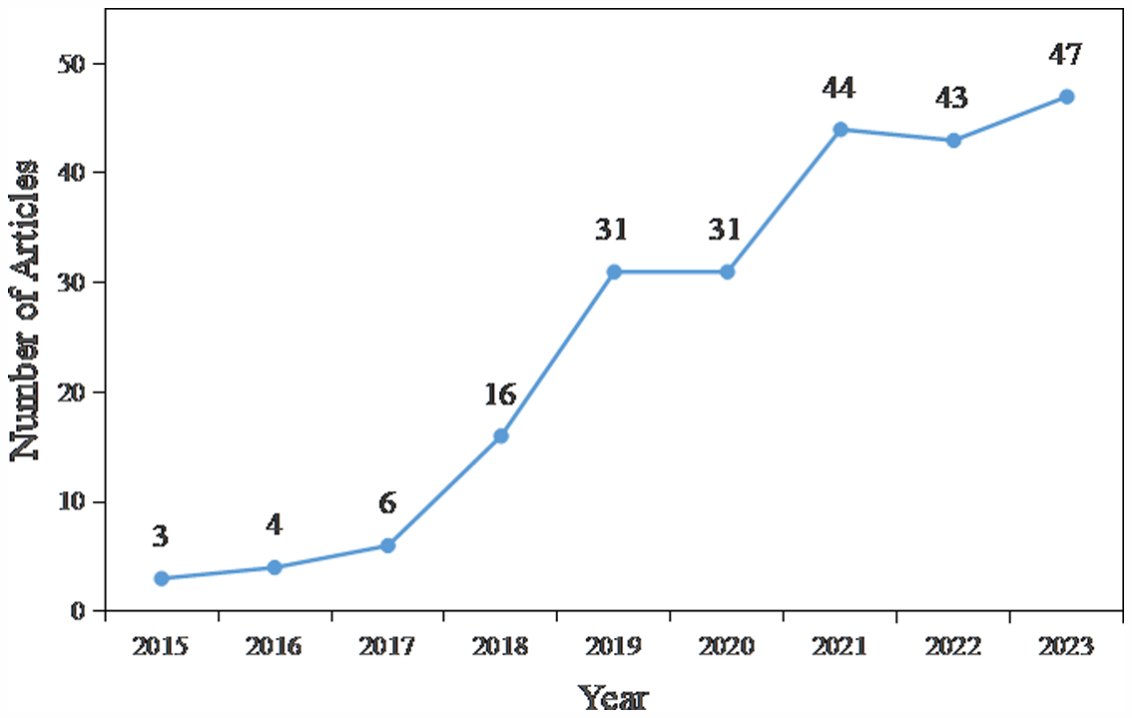
Number of articles.

The collected studies were analyzed by year of publication, as shown in Figure 2. The overall trend of research on emerging mobility services is increasing year by year, with a relative decrease in 2020 due to the COVID-19 pandemic, which had an impact on research in the transport sector. At the same time, it can be seen that research on this topic will continue to be a hot topic in the future.

To facilitate a more precise analysis of these papers, their core elements were extracted, encompassing publication year, titles, and abstracts. The emphasis on titles and abstracts was chosen because these two components typically provide essential information about the research, including its objectives, methods, data sources, and key findings. A meticulous analysis of these core elements enabled a deeper understanding of the research content and contributions of each paper. Furthermore, combining publication years with these core elements allowed for a more comprehensive analysis of topic evolution and trends. By comparing research outcomes from different years, changes and developments in research themes were revealed, providing insights into the forefront dynamics of the research domain.

This data collection exercise established a strong foundation for subsequent data processing and aided in identifying avenues for further investigation through a comprehensive understanding of the literature.

### 2.2 Data processing

Within the data processing module, preliminary data wrangling was performed on the collected research data to prepare it for subsequent training of the LDA model. Initially, the title and abstract content of each document were consolidated into a single text, amalgamating critical information from each document. Subsequently, a stop-word set provided by the Natural Language Toolkit (NLTK) was applied to remove common, non-substantive words such as “and” and purely numeric terms, thus reducing noise and interference.

To ensure uniformity of text format, all uppercase letters were converted to lowercase to mitigate potential issues arising from inconsistent casing, which could lead to word duplication or errors in later analysis. Furthermore, a lemmatizer function was applied to reduce plural forms of words to their base forms. To identify specific word combinations, the text grouped words into bi-grams and tri-grams. Composite words that appeared at least five times in the text were retained and added to the documents in which these combinations occurred, thus extracting richer semantic information.

Through these data processing steps, a cleansed, standardized, and processed textual dataset was obtained. This dataset served as a reliable foundation for subsequent topic modeling and analysis, enabling a more profound understanding of the topics and trends within the textual data.

### 2.3 LDA topic modeling

**Figure 3:**
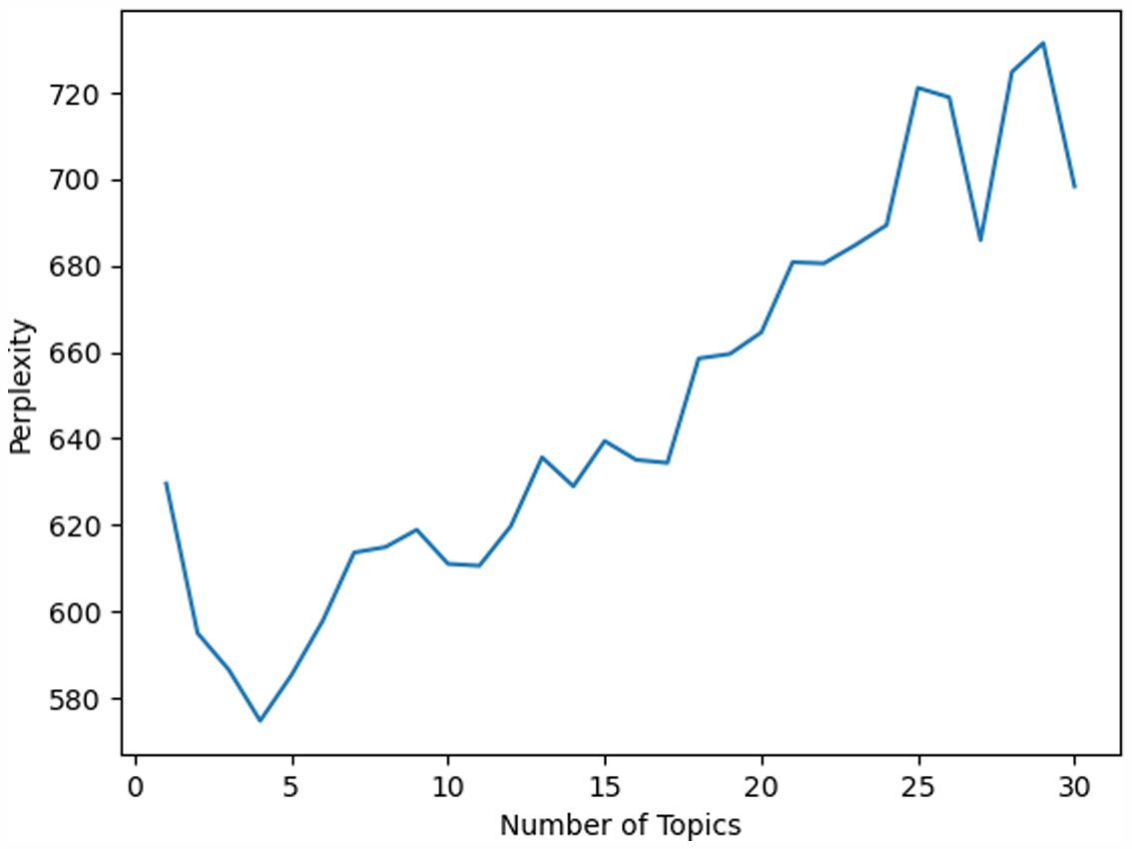
Perplexity scores.

In this study, the LDA model was employed for topic modeling and analysis, aiming to uncover the thematic structure within the emerging mobility services domain in the context of carbon emissions. The training of the LDA model was achieved by maximizing the likelihood function. The objective was to identify a set of topic-word distributions and document-topic distributions that maximized the probability of the observed textual data. For this research, the Sci-Kit Learn package in Python was utilized to implement the training and analysis of the LDA model. To determine the optimal number of topics, perplexity was employed as the evaluation metric. Perplexity is a critical indicator of topic model performance, with lower perplexity values signifying better clustering of the model. As depicted in Figure 3, plotting perplexity scores for varying numbers of topics indicated that the LDA model achieved the lowest perplexity when configured with four topics. With an increase in the number of topics, perplexity continued to rise. As a result, four topics were determined to be the optimal choice.

Upon completing the training, the pyLDAvis package in Python was utilized to visualize the results of the LDA model, thus evaluating the quality of topic categorization. Through pyLDAvis, factors such as the interrelation between topics, key terms associated with each topic, and the distribution of documents within the topic space could be intuitively observed. These visualizations provided valuable insights into the thematic structure of the emerging mobility services domain, revealing the evolution and significant features of topics in the context of carbon emissions. Such insights are crucial for comprehending development trends, critical issues, and sustainability impacts within the realm of emerging mobility services.

### 2.4 Topic intensity

Topic intensity measures the level of attention that researchers allocate to a specific topic. Higher topic intensity signifies greater emphasis on that topic (20). Typically, topic intensity is represented as the sum of probabilities of a topic’s appearance in documents, relative to the total number of documents. A higher topic intensity indicates greater importance of a topic within the entire corpus, reflecting higher research interest. To calculate the intensity value for each topic, the following formula was employed:


$$\theta_{j}=\underset{d}{\sum }\theta_{j}^{\left(d\right)}/M$$

In the formula, where $\theta_{j}$ represents the topic intensity of the j−th topic, reflecting the importance of topic j in the entire document collection, $\theta_{j}^{\left(d\right)}$ represents the probability of topic j appearing in document d, and M is the total number of documents.

In the research domain of emerging mobility services within the context of carbon emissions, prominent research topics tend to receive greater attention, consequently demonstrating relatively higher proportions. Consequently, their topic intensity values are also higher. To gain insight into the level of attention devoted to each topic within the entire corpus, topics with topic intensity values surpassing the average were screened. These topics can be considered as “hot topics,” aiding in the identification of cutting-edge research within the context of emerging mobility services in the context of carbon emissions. Utilizing the topics identified through the LDA model, the core information from 225 papers spanning the years 2015 to 2023 was segmented into eight distinct time intervals. Subsequently, the topic intensity values for different topics within each time interval were computed, revealing the trends in topic intensity evolution across the literature.

## 3 Results

### 3.1 Topic visualization

**Figure 4:**
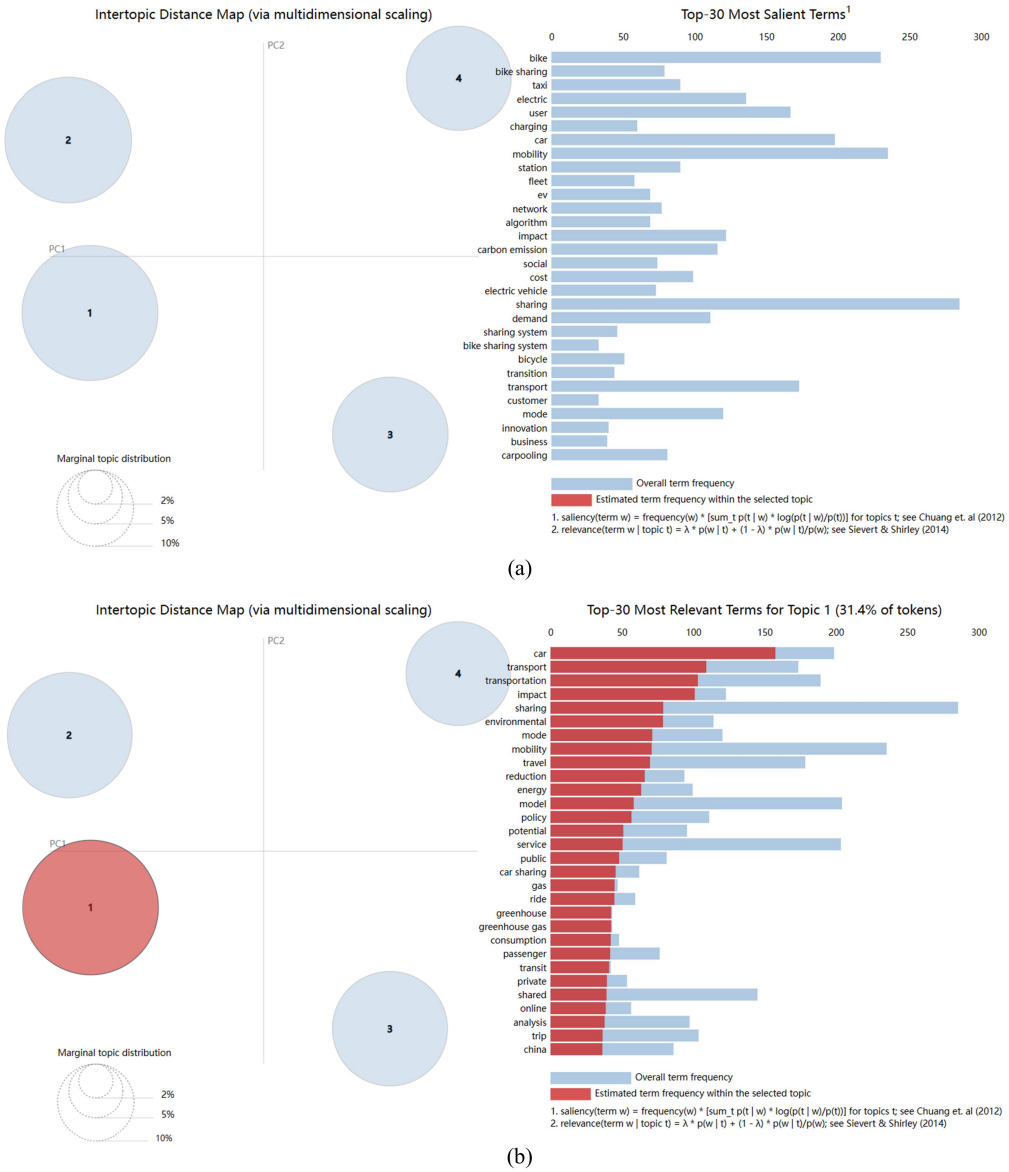
**(a)** Visualization of topic results; **(b)** Visualization of Topic 1.

To facilitate an understanding of the interrelations between topics and the core content of each, an overview of the meanings of the topics was derived from keyword clustering. Utilizing the pyLDAvis package in Python, visual representations of the topic modeling results were generated. Figure 4A illustrates the visual depiction of the topic modeling. On the left side of Figure 4A, four circles correspond to the four topics. The size and positions of these circles, respectively, reflect the frequency and similarity of these topics (21), indicating effective topic classification. When a specific topic is selected, the right side of the figure presents a chart comprising 30 horizontal bars representing the 30 most crucial terms for elucidating that topic. The bars colored in red signify the terms’ weights. Figure 4B provides the visualization results for Topic 1.

**Figure 5:**
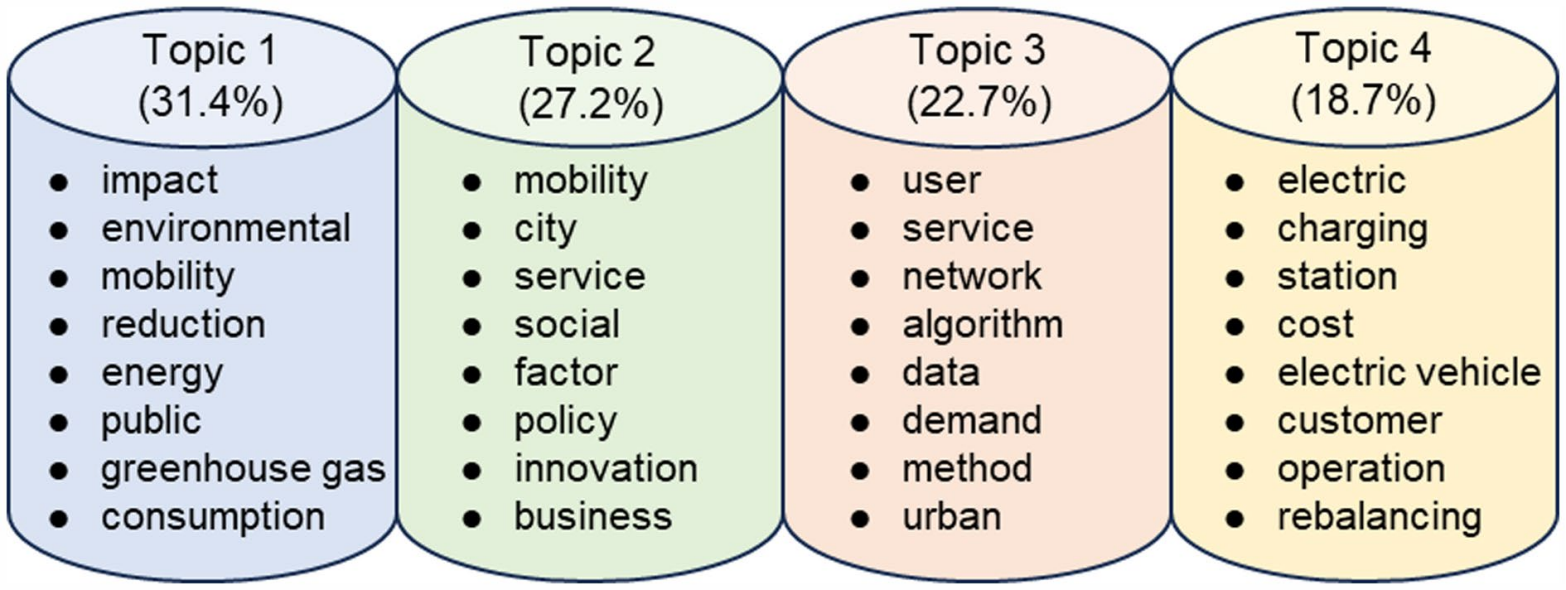
Topic labels.

The topics derived from the LDA model represent probability distributions across the vocabulary. Labels for the four topics pertaining to research on emerging mobility services in the context of carbon neutrality have been summarized using visualizations generated via the pyLDAvis package. These labels are ordered from left to right, in descending order of their probability distribution, as illustrated in Figure 5. The labeling of each topic adhered to the following principles: Firstly, the top 30 terms with the highest frequency from the visualization results for each topic were selected. Secondly, terms that did not contribute significant incremental information were excluded. For instance, terms like “sharing,” “car,” and “transport” were excluded from the analysis due to their lack of substantial information in distinguishing specific topics. Thirdly, representative terms were chosen for words conveying similar meanings. For example, “city” was selected as it adequately represents terms like “city” and “urban,” given their similar connotations.

These four topics encapsulate research on emerging mobility services within the context of carbon emissions over the past 8 years. The development of emerging mobility services is analyzed from the perspectives of the environment, government, consumers, and enterprise providers (22). Additionally, theme labels are incorporated, with each theme named as follows: “Emerging Mobility’s Environmental Impacts” (Topic 1), “Policy-Led Sustainable Mobility Services” (Topic 2), “User-Centric Mobility Services” (Topic 3), and “Cost–Benefit Analysis of Electrification” (Topic 4).

### 3.2 Topic analysis and summary

**Figure 6:**
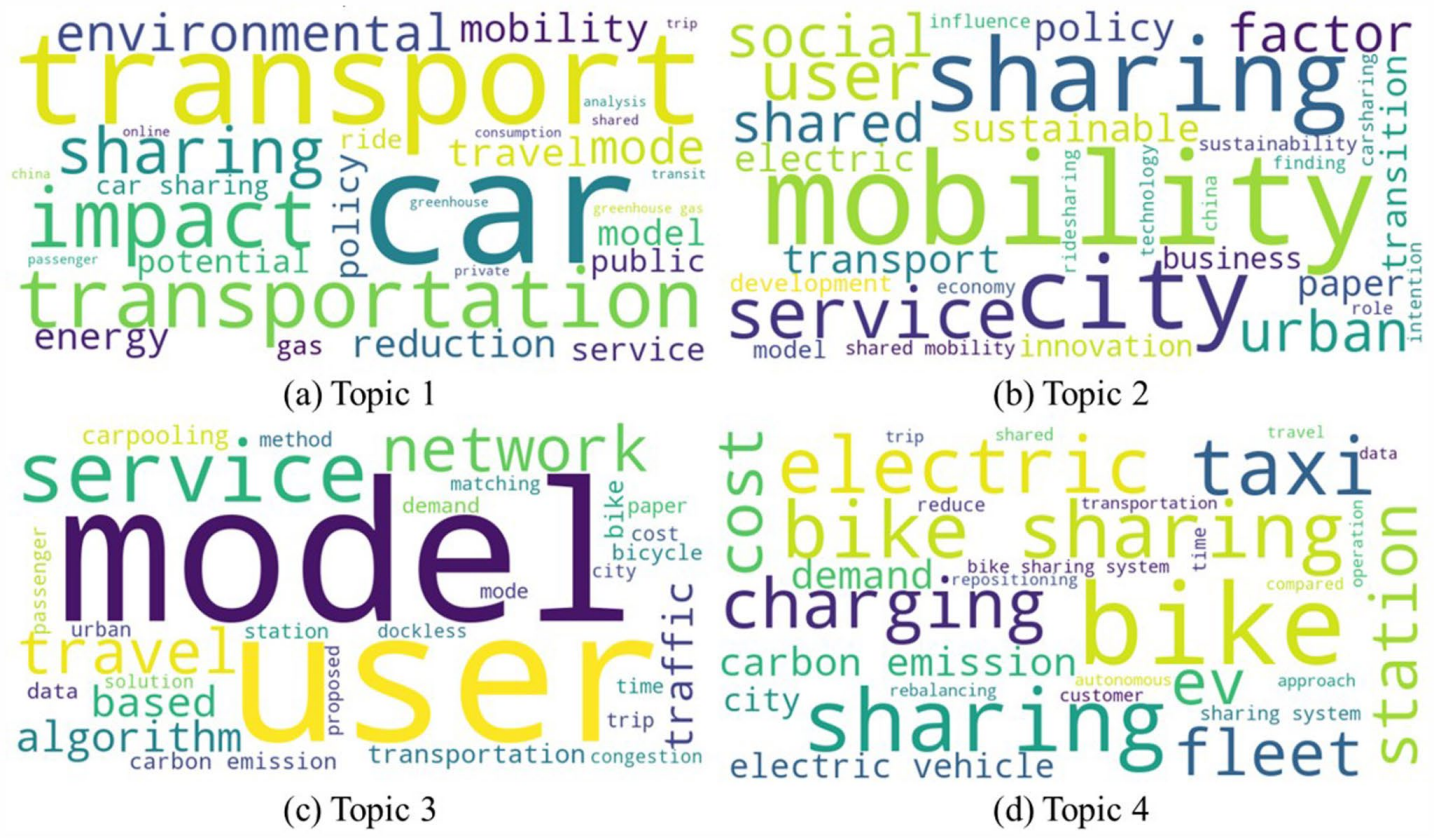
Word cloud for **(a)** Topic 1; **(b)** Topic 2; **(c)** Topic 3; **(d)** Topic 4.

Building upon the topic labels introduced in Section 3.1 and combining them with articles corresponding to each topic in the literature-topic distribution, this section conducts an analysis and summary of research within this domain. The following four key areas of focus are highlighted, and word cloud visualizations are used to present the research emphases of each topic more intuitively. In the word clouds, words appear in varying font sizes and colors, with larger fonts indicating higher word frequency, aiding readers in better understanding the key contents of each topic. Word cloud visualizations for the four topics are shown in Figure 6.

#### 3.2.1 Topic 1: emerging Mobility’s environmental impacts

In “Topic 1: Emerging Mobility’s Environmental Impacts,” the research is focused on examining the environmental consequences of various emerging mobility services. Amatuni et al. (23) discovered that engagement in car-sharing leads to a reduction in annual greenhouse gas emissions associated with mobility. It’s important to note, however, that the impact of this model may vary among individuals (24). When car-sharing replaces personal vehicles, carbon footprints can be reduced (25), and simultaneously, the marginal cost of carbon abatement can decrease (26). Furthermore, promoting ride-sourcing can reduce carbon emissions stemming from decreased car production (27), and large-scale ride-pooling implementation can significantly promote sustainable transportation (28).

For short urban trips, using shared bicycles can effectively reduce carbon emissions and gasoline consumption (29), and optimizing rebalancing operations can maximize emission reductions (30). Sui et al. (31) found that compared to taxis, ride-hailing can effectively reduce idling fuel consumption and lower carbon emissions.

Increasing the number of electric vehicle charging stations in the car-sharing market has been shown to boost electric vehicle adoption, subsequently reducing carbon dioxide emissions (32). However, the potential for greenhouse gas emissions reduction is closely related to the type of vehicles being replaced. For example, Gebhardt et al. (33) found that e-scooters can reduce carbon dioxide emissions when replacing traditional cars, although the effect may differ when replacing battery electric vehicles. A comparative analysis conducted by Ercan et al. (34) underscored the significant reductions in overall transportation emissions when adopting autonomous electric vehicles.

Research in this topic aims to explore the impact of emerging mobility services on the environment, including carbon emissions reduction and the promotion of sustainable transportation. Studies reveal that emerging mobility services such as shared mobility, electric vehicles, and ride-sharing can significantly reduce greenhouse gas emissions while also demonstrating cost-effectiveness on economic and societal levels. Specifically, service providers can adopt advanced algorithms to optimize travel routes, minimize idle time, and reduce unnecessary mileage. Facilitating carpooling services through dynamic ride-matching systems can further enhance vehicle occupancy rates, thereby lowering per capita emissions. However, research also highlights the complexity of individual differences and environmental factors influencing the effects of these services, necessitating further in-depth research to better understand and optimize these impacts.

#### 3.2.2 Topic 2: policy-led sustainable mobility services

In “Topic 2: Policy-Led Sustainable Mobility Services,” multiple research studies delve into the influential role of policies in steering the development of sustainable mobility services. Luna et al. (35) discovered that incentive policies for Brazil’s inaugural electric vehicle sharing program significantly reduced carbon emissions and bolstered electric vehicle adoption. To promote shared electric vehicles effectively, policies must be finely tuned to different demographic groups in varying regions (36). Similar tailored approaches are vital for accelerating shared bicycle adoption (37). The influence of community norms looms large when shaping policies for electric vehicle access (38). Tikoudis et al. (39) reported that carbon emissions from shared mobility services decrease with robust policy support but exhibit significant variation among different cities.

During the COVID-19 pandemic, Zhang et al. (40) observed a correlation between the pandemic’s severity and increased use of car-sharing and bike-sharing services. In periods of lower pandemic severity, shared mobility aids in reducing carbon emissions, necessitating a delicate balance between promoting sustainable shared mobility and pandemic control. Lan et al. (41) underscored the significance of societal, business, and government roles in advancing sustainability within the sharing economy. Empirical investigations by Ma et al. (42) shed light on the co-evolution of urban sustainability and the innovation of green intelligent transportation business ecosystems. Sherriff et al. (43) highlighted the need for shared bicycle operators to consider a city’s social and natural geographical environment more closely and collaborate more effectively with transportation management agencies and local governments to alleviate congestion and promote low-carbon travel. Government regulation and carbon emission reduction certification significantly enhance user willingness to carpool, while information dissemination also encourages carpooling behavior (44). Policymakers and operation designers can leverage these findings to craft more effective marketing strategies for promoting carpooling and realizing greater financial and environmental benefits (45).

Research in this domain underscores the pivotal role played by policies in leading the development of emerging sustainable mobility services. This role encompasses policy formulation, region-specific strategies, social role evolution, and collaborative innovation. Specific policies that governments can adopt include environmental taxes (46), subsidies (47), regulatory measures (48), emissions trading scheme (49), and other related strategies. However, governments must delicately balance the promotion of sustainable transportation with their response to emergencies. These studies provide valuable insights for urban planning and policymaking, fostering more eco-friendly and sustainable urban transportation systems.

#### 3.2.3 Topic 3: user-centric mobility services

In “Topic 3: User-Centric Mobility Services,” the research focuses on user-oriented mobility services. Its primary objective is to explore how to meet evolving user demands while advancing environmental preservation and operational efficiency.

Due to the inherent uncertainty in user travel demands, operators of shared mobility systems aim to enhance user services by accurately predicting service needs, facilitating rational vehicle allocation. For instance, Zhang et al. (50) proposed a dynamic bicycle repositioning method for public bicycle sharing systems, optimizing vehicle allocation routes. Similarly, Zhou et al. (51) developed short-term demand forecasting models for shared bicycles. Wang et al. (52) created micro-demand forecasting models for one-way electric vehicle sharing systems, enabling intelligent vehicle scheduling and promoting eco-friendly, low-carbon travel. Peak morning hours witness the highest public transportation usage. To address the uneven distribution of shared bicycles along subway lines during these hours, Zhang et al. (53) proposed an optimal low-carbon allocation scheme connecting subway routes. In addition to residents’ daily commutes, Buning and Lulla (54) researched tourists’ travel behaviors to enhance the tourist experience. To safeguard user privacy during carpooling and prevent personal information leakage, Li et al. (55) designed privacy-focused carpooling schemes.

To better match passengers with drivers, Liu et al. (56) introduced a method estimating carpooling travel time and psychological costs by considering the motivations and preferences of potential carpoolers. Guo et al. (57), recognizing the dynamic nature of real-world environments, proposed a real-time ride-sharing framework with dynamic time windows and expected migration-based mechanisms. Hua et al. (58) delved into the dynamic shared taxi problem within on-demand ride-sharing systems. Addressing the environmental conservation vs. matching rates paradox, Ma et al. (59) developed a carpooling service model to avoid potential mismatches and increased carbon emissions due to time and efficiency losses. Carpooling also has the potential to promote social integration (60). In an effort to understand the behavioral preferences of online ride-hailing consumers, Lin et al. (61) used text sentiment analysis on app review data to optimize services. Furthermore, to enhance the utilization of electric vehicles, Zhang et al. (62) optimized vehicle allocation and relaying within shared systems, considering user travel distances.

This topic focuses on improving the intelligence, user experience, and environmental sustainability of mobility services. Research encompasses travel demand prediction, user privacy protection, carbon emissions reduction, and user experience enhancement. The topic emphasizes the importance of leveraging technological innovations to cater to diverse user needs while prioritizing environmental conservation and privacy security, offering intelligent and sustainable solutions for urban mobility.

#### 3.2.4 Topic 4: cost–benefit analysis of electrification

In “Topic 4: Cost–Benefit Analysis of Electrification,” the research predominantly centers around conducting cost-effectiveness analyses of electric vehicles (EVs), with a particular emphasis on their potential to reduce carbon emissions and enhance energy efficiency.

Electric vehicles are known for their energy efficiency and reliability, especially when combined with renewable energy sources. This synergy significantly contributes to the reduction of carbon emissions. The effectiveness of emissions reduction in shared autonomous electric vehicles (SAEVs) hinges on several factors, including fleet expansion, operational strategies, and their interactions with the power system. Jones and Leibowicz (63) observed that widespread adoption of SAEVs can lead to cost reductions and substantial reductions in carbon emissions. Taking a fleet operator’s perspective, Loeb and Kockelman (64) provide a detailed estimation of the costs associated with SAEV fleets, revealing that electric vehicles may not exhibit significant economic advantages in the short term but offer substantial environmental benefits.

Electric vehicles do face challenges, such as relatively shorter driving ranges and longer charging times. Recognizing the growing demand for electric vehicle charging infrastructure, Zhang et al. (65) conducted an economic and environmental impact assessment, shedding light on the influence of electrification on urban transportation. Iacobucci et al. (66) optimized SAEV operations, resulting in reduced charging costs and carbon emissions. For electric vehicle fleets to maximize revenue, dynamic optimization of trip pricing and electric vehicle dispatch decisions is essential (67). Aligning charging schedules with renewable energy generation emerges as a critical determinant of the economic and emissions impact of SAEV fleets (68).

The widespread adoption of ride-hailing mobile applications has facilitated the computerization and optimization of taxi service strategies. To encourage taxi drivers to transition to electric taxis, Tseng et al. (69) evaluated the impact of different battery capacities and charging conditions on the profitability of electric taxi drivers. In an effort to minimize average trip times and charging costs for all customers, Ammous et al. (70) investigated strategies to reduce the inconvenience caused by in-route customer charging and operator charging station placement. Additionally, Dong et al. (71) found that businesses and government-related agencies can leverage the cost advantages of shared cars to promote low-carbon and green development (72).

This topic underscores the potential of EVs in reducing carbon emissions and enhancing energy efficiency, particularly through the widespread adoption of SAEVs. While EVs may face economic challenges in the short term, their environmental benefits are significant. Research also emphasizes the optimization of operations, dynamic pricing, alignment with renewable energy sources, and the electrification transformation of the taxi industry. These factors are critical in unlocking the potential of electric vehicles.

### 3.3 Topic intensity analysis

**Figure 7:**
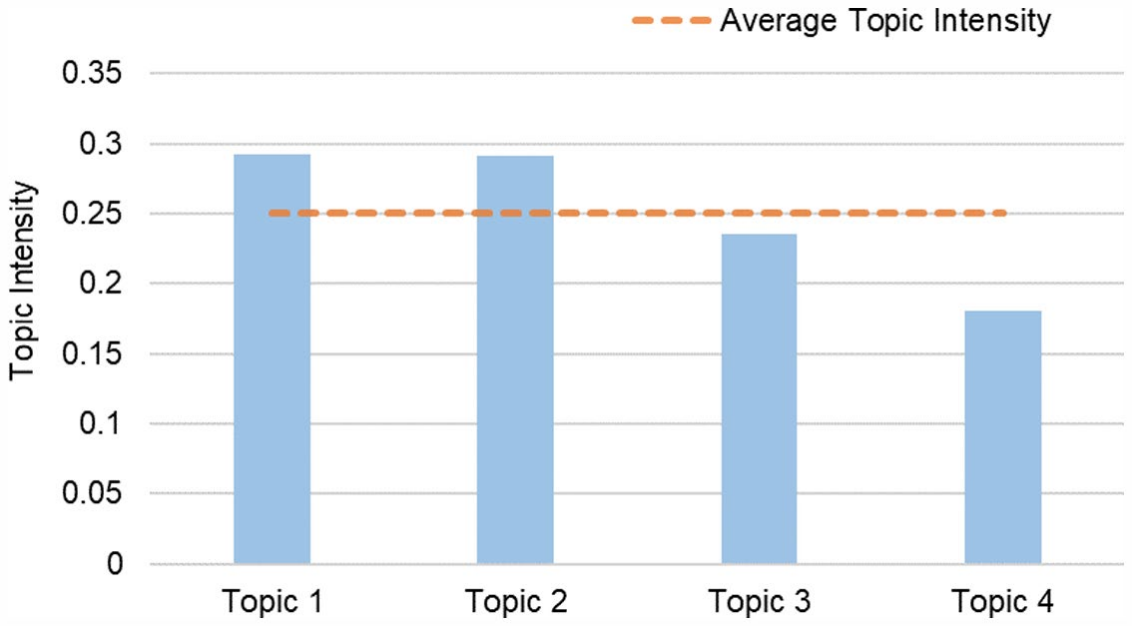
Topic intensity.

Following the identification of the topics, calculations were performed to determine their respective intensities using a formulated formula. These results have been visually represented in Figure 7, where the height of each bar corresponds to the numerical value of the topic intensity. The central horizontal line in the figure represents the average topic intensity value.

Upon analyzing the data presented in Figure 7, it becomes evident that Topic 1 and Topic 2 exhibit significantly higher topic intensities compared to the average level. The signing of the Paris Agreement in 2015 marked a new level of global consensus on energy conservation and emission reduction. Since then, countries worldwide have responded by introducing a series of policies and measures aimed at promoting energy conservation and reducing greenhouse gas emissions. The implementation of these policies has not only established a solid foundation for environmental protection and climate change mitigation but has also fostered the development and progress of these two areas. It highlights the growing attention from both researchers and policymakers towards the environmental effects and the governmental and societal actions related to emerging mobility services. These two topics have the potential to significantly impact both academic research and real-world applications.

Concurrently, Topic 3’s topic intensity falls slightly below the average level, signaling that researchers and practitioners express relatively heightened interest in the realm of user services within emerging mobile services. This increased interest likely stems from the paramount importance of user experience and demand in determining the overall success of these services. While Topic 4 currently exhibits a relatively lower topic intensity, it is conceivable that the potential of electrified transportation may receive greater recognition and research attention in the future, owing to society’s growing demand for sustainable transportation solutions.

### 3.4 Topic evolution over time

**Figure 8:**
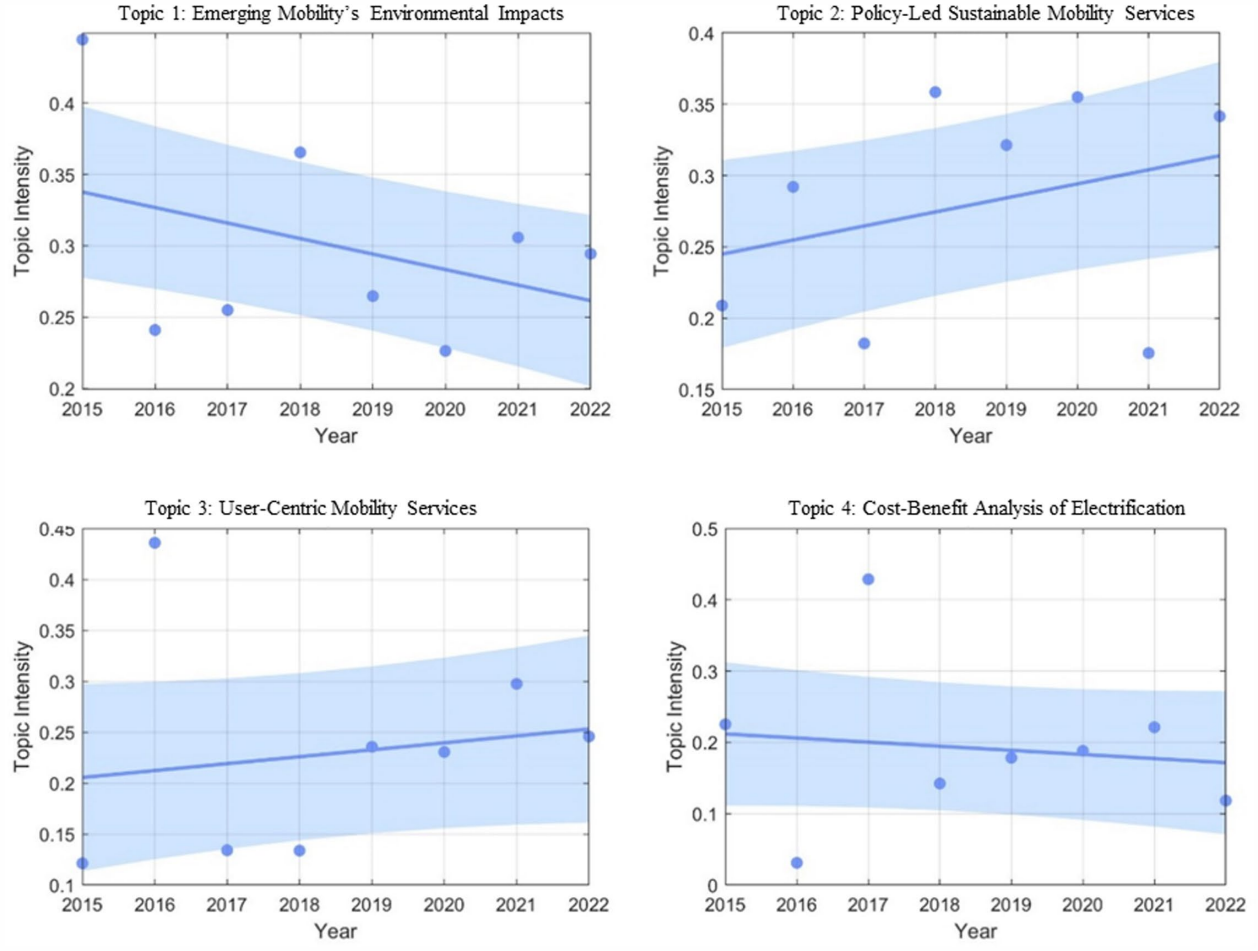
Topic intensity over time.

From 2015 to 2023, variations in the intensities of the identified topics were meticulously tracked. The outcomes are visually presented in Figure 8. Due to the limited availability of data points, it presented a challenge to pinpoint specific trends. Consequently, regression lines were employed to graphically depict these trends.

As portrayed in Figure 8, Topic 2 and Topic 3 exhibit upward trends, while Topic 1 displays a declining trend, and Topic 4 maintains a relatively stable trajectory. The ascending trend observed in Topic 2 can be attributed to sustained engagement and backing from governmental and societal institutions in the sphere of emerging mobile services (73, 74). This phenomenon is particularly conspicuous in the formulation of policies and initiatives aimed at curtailing carbon emissions and augmenting the sustainability of urban transportation, echoing the findings of Zhang et al. (75).

The ascending trajectory in Topic 3 likely mirrors the ardent pursuit by operators in the domain of emerging mobile services to cater to user demands and enhance user experiences, driven by intense market competition (76). Faced with cutthroat competition, these service providers are compelled to offer more astute, efficient, and user-centric solutions to captivate a broader user base.

Although Topic 1 maintains an overall high intensity but demonstrates a diminishing trend, this trend can be attributed to the adoption of the Paris Agreement and global climate action strategies (77) subsequent to the 2015 United Nations Climate Change Conference. Diverse nations and organizations are progressively striving to attain their respective “carbon peaks” and “carbon neutrality” objectives (78–81). The inception and implementation of worldwide policies may incite extensive climate action (82), positioning research into environmental impact to adapt progressively in support of these global imperatives. This shift, however, does not imply a diminished emphasis on environmental concerns; rather, it signifies the diversification of research within the field.

Lastly, Topic 4 exhibits a relatively consistent trajectory. Nevertheless, the advocacy for electrified transportation remains pivotal for curbing automobile emissions, mitigating fossil fuel dependency, augmenting energy efficiency, and propelling the entire transportation sector towards a more sustainable future (83). The stability of this trend likely reflects the enduring robustness of the electrified transportation domain (84), which is indispensable in tackling challenges pertaining to climate and energy.

## 4 Discussion

### 4.1 Main research findings

Through a comprehensive examination of the literature and an analysis of thematic evolution, this investigation explored critical issues within the realm of emerging mobility services concerning carbon emissions, resulting in the following principal findings:

LDA unveiled four core research topics: “Emerging Mobility’s Environmental Impacts,” “Policy-Led Sustainable Mobility Services,” “User-Centric Mobility Services,” and “Cost–Benefit Analysis of Electrification.”

Emerging mobility services possess the potential to mitigate carbon emissions and enhance urban air quality. Existing research suggests that they could positively impact the environment and contribute to the development of sustainable transportation. However, the extent of this impact varies based on regional, service type, and individual factors. Topic 1 exhibits a high level of thematic intensity, but a declining trend over time. This partly suggests a consensus on the positive environmental impact of emerging mobility services, with a more mature theoretical framework in place. It also indicates that the promotion of these services aligns with global energy-saving and emission-reduction goals.

Governmental bodies play a pivotal role in fostering low-carbon development in emerging mobility services through incentive policies and region-specific strategies. Collaborative evolution across societal, commercial, and governmental roles is instrumental in advancing sustainable transportation. Topic 2 shows an upward trend. This highlights the need for governments and policymakers to closely monitor research on emerging mobility services to inform decision-making based on the latest findings.

Innovations in transportation services lead to more intelligent, efficient, and sustainable travel solutions, meeting evolving user demands and achieving environmental and efficiency benefits. Topic 3 also shows an upward trend, which necessitates that service providers balance user needs with business interests. Meeting user needs and attracting a broader user base would positively impact business outcomes.

Despite facing short-term economic challenges, electric vehicles offer significant environmental benefits, especially when optimized for fleet scale, operational efficiency, and integration with renewable energy sources. Topic 4 exhibits a relatively low thematic intensity and a flat trend. This may be due to the fact that, while electrification could offer environmental benefits, it incurs higher costs for service providers. Therefore, policymakers may consider offering certain policy incentives to service providers to maximize the role of electrification in sustainable transportation.

Overall, the promotion of emerging mobility services could support the goals of energy conservation, meet user needs, and foster business development. However, this requires the cooperation of all relevant stakeholders. For example, governments must formulate policies and strategies to support enterprises in providing these services; enterprises must aim to maximize user satisfaction while delivering the services; and users should consider the societal and environmental impacts when utilizing these services, thus contributing to a virtuous cycle that drives the development of sustainable transportation.

### 4.2 Future research directions

Given the imperative goal of achieving carbon emission reduction in emerging mobility services, the following four key research directions are proposed:

Synergistic Optimization of Emerging Mobility Services and Energy Systems: Focus on optimizing the synergy between emerging mobility services and energy systems to minimize carbon emissions and enhance system efficiency. Employ a multidisciplinary approach, integrating intelligent electric vehicle charging strategies, dynamic renewable energy management, power grid resilience, and sustainable urban planning to aid decision-makers in achieving optimal carbon emission reduction and resource utilization.

Understanding the Correlation between Carbon Footprints and User Behavior: Conduct comprehensive analysis of user behavior (85) using sophisticated techniques such as big data analysis, machine learning algorithms, behavioral economics models, and artificial intelligence technologies (86). Identify critical factors influencing user travel choices to enable effective guidance towards sustainable travel modes and reduction of carbon emissions.

Collaborative Development of Urban Planning and Transportation Infrastructure: Adjust urban planning and transportation infrastructure to accommodate the proliferation of shared mobility services and electric vehicles. Leverage emerging technologies like Benders decomposition algorithm (87), intelligent transportation systems, urban simulation techniques, and blockchain to enhance urban sustainability. Simulate various urban design scenarios to evaluate their impact on carbon emissions and travel efficiency, and deploy electric vehicle charging infrastructure to promote sustainable urban transportation.

Implementation of Policy Instruments for Carbon Emission Reduction in Emerging Mobility Services: Explore how governments and regulatory bodies can utilize policy instruments like carbon taxes, trading systems, and incentives for green energy to guide carbon emission reduction in the emerging mobility services sector. Utilize mathematical modeling and policy analysis to assess the impacts of different policy tools, and encourage international cooperation to share best practices and technical knowledge, facilitating countries in achieving carbon neutrality goals.

## 5 Conclusion

Topic modeling is a valuable tool for identifying research themes in the realm of carbon emissions in emerging mobile services and for helping researchers understand shifts in research focus by tracking topic intensity. In this study, 225 relevant articles from the Web of Science Core Collection database, covering the period from 2015 to 2023, were retrieved and curated. Utilizing the LDA model, four key topics were identified within these articles. These included “Emerging Mobility’s Environmental Impacts” (Topic 1), “Policy-Led Sustainable Mobility Services” (Topic 2), “User-Centric Mobility Services” (Topic 3), and “Cost–Benefit Analysis of Electrification” (Topic 4). The topic intensity analysis revealed that Topic 1 and 2 are currently the most researched. The study also examined the evolution of these topic over time and found that Topic 2 and 3 are exhibiting an upward trend. This suggests that emerging mobility services will, to some extent, be shaped by policies and user needs in the future. The study concludes by outlining directions for future research.

The study provides valuable insights for researchers, governments, and industry service providers. Based on its findings, scholars could explore additional dimensions, investigate various aspects of emerging mobility services in greater depth, or focus on specific topics to advance their research. The study also offers valuable guidance for government agencies in formulating policies to promote sustainable transportation. Additionally, it provides useful insights for industry service providers to optimize service quality and enhance economic efficiency.

It’s essential to acknowledge potential limitations when interpreting the study’s results. Firstly, this study was limited to the Web of core databases during data collection and did not include literature from other databases, resulting in neglecting the contribution of other literature. Furthermore, the LDA model assumes independence between topics, whereas practical research often reveals interconnections between them. In future research, the Correlated Topic Model (CTM) will be employed to assess thematic relevance and enhance the study’s rigor. Additionally, this study examines the thematic landscape of research on emerging mobility services but does not delve deeply into the underlying concept. Future research could utilize established theoretical frameworks such as the Theory of Planned Behavior (TPB) and Diffusion of Innovations (DOI) to better understand the adoption and optimization of these services. Incorporating these theories may offer a robust conceptual foundation for improving the strategic design and implementation of emerging mobility services.

## Data Availability

The raw data supporting the conclusions of this article will be made available by the authors, without undue reservation.
